# The burden associated with generalized pustular psoriasis: A Canadian population-based study of inpatient care, emergency departments, and hospital- or community-based outpatient clinics

**DOI:** 10.1016/j.jdin.2023.03.012

**Published:** 2023-04-14

**Authors:** Jean-Eric Tarride, Vimal H. Prajapati, Charles Lynde, Gord Blackhouse

**Affiliations:** a Department of Health Research Methods, Evidence and Impact, Faculty of Health Sciences, McMaster University, Hamilton, Canada; b Programs for Assessment of Technology in Health (PATH), The Research Institute of St. Joe’s Hamilton, St. Joseph’s Healthcare Hamilton, Hamilton, Canada; c Center for Health Economics and Policy Analysis (CHEPA), McMaster University, Hamilton, Canada; d Division of Dermatology, Department of Medicine; Section of Community Pediatrics, Department of Pediatrics; and Section of Pediatric Rheumatology, Department of Pediatrics, University of Calgary, Calgary, Canada; Skin Health & Wellness Centre, Calgary, Canada; Dermatology Research Institute, Calgary, Canada; and Probity Medical Research, Calgary, Canada; e Department of Medicine, University of Toronto, Toronto, Canada

**Keywords:** Canada, generalized pustular psoriasis, healthcare expenditures, inpatient mortality

## Abstract

**Background:**

Not much is known about the burden of generalized pustular psoriasis (GPP).

**Objectives:**

To document the burden of GPP in Canada and to compare it with psoriasis vulgaris (PV).

**Methods:**

National data were used to identify Canadian adult patients with GPP or PV hospitalized or visiting an emergency department (ED) or hospital-/community-based clinic between April 1, 2007, and March 31, 2020. Analyses of 10-year prevalence and 3-year incidence were conducted. Costs were determined when the most responsible diagnosis (MRD) was GPP or PV (MRD costs) and for all reasons (all-cause costs).

**Results:**

In the prevalence analysis, 10-year mean (SD) MRD costs were $2393 ($11,410) for patients with GPP and $222 ($1828) for those with PV (*P* < .01). In the incidence analysis, patients with GPP had higher 3-year mean (SD) MRD costs ($3477 [$14,979] vs $503 [$2267] for PV; *P* < .01). Higher all-cause costs were also associated with patients with GPP. Inpatient/ED mortality was higher in the GPP group in our 10-year prevalence (9.2% for patients with GPP vs 7.3% for those with PV; *P* = .01) and 3-year incidence (5.2% for patients with GPP and 2.1% for those with PV; *P* = .03) analyses.

**Limitations:**

Physician and prescription drug data were not available.

**Conclusion:**

Patients with GPP incurred higher costs and mortality than patients with PV.

**Capsule Summary**

• In Canada, patients with generalized pustular psoriasis hospitalized or visiting an emergency department or a hospital-/community-based clinic incur higher costs and inpatient mortality than patients with psoriasis vulgaris.
• Clinicians, patients, and researchers should be aware of the incremental burden of generalized pustular psoriasis and further explore its impact beyond health care expenditures.

## Introduction

Generalized pustular psoriasis (GPP) is a rare subtype of pustular psoriasis, which can occur with or without a history of plaque psoriasis.^1^, ^2^, ^3^ The clinical course of GPP is heterogeneous as it can be either a relapsing disease with recurrent flareups and periods of remission or a persistent disease with intermittent flareups.^1^^,^^2^ Although the severity of signs and symptoms is variable with each flareup for a particular patient, flareups may lead to emergency department (ED) visits or may require inpatient care for patients with extensive cutaneous and/or systemic involvement, as these individuals can be at a higher risk for complications.^1^^,^^2^

Because of its low prevalence (ranging from 0.18 to 12 per 100,000),^4^, ^5^, ^6^, ^7^ the burden associated with GPP has not been well documented in the past.^8^ However, recent evidence from the United States,^9^ Sweden,^10^ and Japan^11^ have shown a higher economic burden for patients with GPP than for patients with psoriasis vulgaris (PV) or the general population. A recent population-based study of ED and inpatient care from the province of Ontario in Canada (13.6 million habitants) identified 215 ED visits and 75 hospitalizations of patients with GPP over a 10-year period.^12^ However, this Canadian study was limited as it did not provide any other information on the characteristics of these ED visits and hospitalizations (eg, length of stay and costs). To better inform Canadian patients, physicians, and health care administrators as well as to contribute new information to the existing literature, the objective of this study was to determine the burden of GPP in Canada among patients with GPP seen in Canadian hospitals, EDs, and hospital-/community-based outpatient clinics and to compare with that of patients with PV.

## Methods

### Study overview and data sources

A Canadian population-based cohort study using existing de-identified patient-level data from 2 databases from the Canadian Institute for Health Information (CIHI)^13^ was conducted to identify all Canadians hospitalized and visiting an ED or hospital-/community-based outpatient clinic for either GPP (GPP cohort) or PV (PV cohort). The first database was CIHI’s Discharge Abstract Database (DAD),^14^ which captures administrative, clinical, and demographic information on all hospital discharges (including deaths) across Canada. The second database was the National Ambulatory Care Reporting System (NACRS),^15^ which depending on the years, captures data on approximately 64% (2016-17) to 85% (2019-20)^16^ of all hospital-/community-based ED visits and ambulatory care visits in Canada. Both DAD and NACRS data from the province of Quebec (approximately 23% of the Canadian population) were not included. Data on inpatient or ED mortality in the facility were available in DAD or NACRS, but data of deaths in the community were not available.

For each encounter captured in CIHI DAD and NACRS data, up to 20 possible International Statistical Classification of Diseases and Related Health Problems, Tenth Revision, Canada (ICD-10-CA) codes are recorded and categorized as the “most responsible diagnosis (MRD)” (ie, the diagnosis determined to have been responsible for the greatest portion of the patient’s length of stay) or as another diagnosis/comorbidity. In addition, health care expenditures (ie, costs) associated with each encounter are available based on the CIHI costing methodology.^17^ The 2 databases were linked to each other using the CIHI Meaningless but Unique Patient Identifier, which is common to the 2 databases.

### Study population

The study population included Canadian adults (≥18 years of age) hospitalized or visiting an ED or hospital-/community-based outpatient clinic between April 1, 2017, and March 31, 2020, with a diagnostic code indicating GPP or PV. ICD-10-CA codes of L40.1 were used to define the GPP cohort, whereas ICD-10-CA codes of L40.0 or L40.9 were used to define the PV cohort. Exclusion criteria included diagnostic codes indicating dermatitis due to substances taken internally (ICD-10-CA code: L27 category, which includes generalized or local skin eruption due to drugs and medicaments taken internally) or acrodermatitis continua (ICD-10-CA code: L40.2). Prevalence and incidence approaches were used.

### Prevalence analysis

Ten-year prevalence was estimated based on the number of unique patients with at least 1 DAD or NACRS record between April 1, 2010, and March 31, 2020, with a diagnostic code indicating GPP for the GPP cohort and PV for the PV cohort. Prevalent cases were described in terms of age, sex, medical history, and comorbidities commonly reported in the literature (eg, psoriatic arthritis, diabetes, hypertension, hyperlipidemia, myocardial infarction, stroke, cigarette smoking, obesity, anxiety, depression, atopic dermatitis, gout, thyroid disorders, and sleep apnea).^9^^,^^11^^,^^18^ Ten-year health care resource utilization, costs, and all-cause inpatient/ED mortality were estimated based on DAD and NACRS data.

### Incidence analysis

Incident cases were defined as unique patients with a GPP or PV diagnostic code over a 5-year time period (April 1, 2012, to March 31, 2017) without any GPP (GPP incident cohort) or PV (PV incident cohort) diagnostic code in the previous 5 years (April 1, 2007, to March 31, 2012). As such, incident cases have a minimum of 3 years of follow-up (up to March 31, 2020). To ensure that the index event (eg, hospitalization, ED visit, or outpatient clinic visit) was directly attributable to GPP or PV and not to an unrelated condition, 5-year incident cases of GPP and PV were identified as the first DAD or NACRS record with an MRD of GPP and PV, respectively. Patient characteristics at the time of index and 3-year health care resource utilization, costs, and all-cause inpatient/ED mortality following the index event were documented using CIHI data.

### Statistical analysis

Prevalence and incidence rates were estimated by dividing the number of prevalent/incident cases meeting our inclusion criteria by the Canadian population estimates (except Quebec) during the same time period. In addition to descriptive statistics, student *t* tests and χ^2^ tests were used to compare the baseline characteristics of the GPP and PV populations. Several analyses were conducted to present the epidemiological and economic results. Since reporting information in NACRS is only mandatory in Ontario, Alberta, and Yukon, the prevalence and incidence based on all Canadian provinces/territories may underestimate the number of prevalent and incident cases of GPP and PV. Therefore, sensitivity analyses were conducted using Ontario data only (38% of the Canadian population; 100% reporting of ED visits) and Alberta data only (12% of the Canadian population; 100% reporting of ED visits and 100% reporting of hospital-/community-based clinic visits) to determine the prevalence and incidence rates. Two analyses were conducted to document the economic burden of GPP and PV. First, health care resource utilization and costs directly attributable to GPP or PV were documented by identifying DAD and NACRS records for which the MRD (ie, diagnosis determined to have been responsible for the greatest portion of the patient’s length of stay) was GPP or PV (ie, MRD costs). The second set of analyses considered all health care resources consumed by our study populations independent of the presence of a GPP/PV diagnostic code (ie, all-cause costs).

The mean number of admissions, length of stay, and costs between the GPP and PV cohorts were compared using multivariable generalized linear models (log link with negative binomial distribution for count data and gamma distribution for costs) adjusting for baseline characteristics (age, sex, and comorbidities). Inpatient/ED mortality between GPP and PV cohorts was compared using multivariable logistic regressions adjusting for baseline characteristics. To comply with CIHI requirements, cells for which 1 to 4 individuals contributed to the data were not disclosed. All costs are expressed in Canadian dollars unless otherwise stated.

## Results

### Prevalence analyses

Over the 10-year period from April 1, 2010, to March 31, 2020, a total of 607 and 24,828 individuals with a GPP and PV record were respectively identified in DAD and NACRS. The corresponding prevalence rates were 2.77 per million individuals for GPP and 113.13 per million individuals for PV. Sensitivity analyses of our study indicated that prevalence rates were similar in Ontario (GPP: 2.72 per million individuals; PV: 114.69 per million individuals) where 100% of ED visits are reported, and higher in Alberta (GPP: 5.44 per million individuals; PV: 272.85 per million individuals) where 100% of ED visits and hospital-/community-based clinic visits are captured.

As shown in Table I, there was a higher proportion of women with GPP (54.5%) compared with that of women with PV (44.1%) (*P* < .01). The mean (SD) age was similar between GPP (53.1 [17.0] years) and PV (53.3 [18.3] years) prevalent cases (*P* = .75), with more than 70% of the population between the ages of 18 and 65 years. Approximately 50% of our cohorts were from Ontario. With the exception of depression (8.4% for GPP cohort vs 11.3% for PV cohort; *P* = .02) and psoriatic arthritis (4.3% for GPP cohort vs 2.2% for PV cohort; *P* < .01), there were no differences in comorbidities between the cohorts.

**Table I:** Prevalence analysis: baseline characteristics

Characteristic	GPP N = 607 (%)	PV N = 24,828 (%)	*P* value
Women	331 (54.5)	10,954 (44.1)	<.01
Mean age (SD)	53.1 (17.0)	53.3 (18.3)	.75
Age categories (y)			.06
18-25	10 (3.1)	888 (6.8)	
26-35	70 (11.5)	3155 (12.7)	
36-45	94 (15.5)	3429 (13.8)	
46-55	130 (21.4)	4466 (18.0)	
56-65	123 (20.3)	4977 (20.0)	
66-75	97 (16.0)	3852 (15.5)	
≥75	62 (10.2)	3191 (12.9)	
Province/Territory			<.01
Alberta	172 (28.3)	8633 (34.8)	
British Columbia	48 (7.9)	910 (3.7)	
Saskatchewan	20 (3.3)	608 (2.4)	
Manitoba	16 (2.6)	580 (2.3)	
Ontario	299 (49.3)	12,609 (50.8)	
Nova Scotia, New Brunswick, Prince Edward Island, Newfoundland, and Territories	52 (8.6)	1488 (6.2)	
Comorbidities			
Hypertension	189 (31.1)	8166 (32.9)	.36
Diabetes	112 (18.5)	5026 (20.2)	.28
Cigarette smoking	63 (10.4)	2115 (8.5)	.11
Hyperlipidemia	38 (6.3)	1946 (7.8)	.15
Myocardial infarction	37 (6.1)	1464 (5.9)	.84
Stroke	21 (3.5)	1060 (4.3)	.33
Obesity	41 (6.8)	1967 (7.9)	.29
Sleep apnea	26 (4.3)	1302 (5.2)	.29
Depression	51 (8.4)	2810 (11.3)	.02
Anxiety	14 (2.3)	819 (3.3)	.17
Thyroid disorders	31 (5.1)	1576 (6.3)	.21
Atopic dermatitis	ND	325 (1.3)	.16
Gout	29 (4.8)	1256 (5.1)	.75
Psoriatic arthritis	26 (4.3)	534 (2.2)	<.01

*GPP,* Generalized pustular psoriasis; *ND,* nondisclosable; *PV,* psoriasis vulgaris; *SD,* standard deviation.

When compared with patients with PV, patients with GPP incurred higher 10-year mean (SD) MRD costs ($2393 [$11,410] vs $222 [$1828]; *P* < .01). Ten-year mean (SD) all-cause costs were $49,695 ($111,582) for GPP cohort and $42,574 ($83,960) for PV cohort (*P* < .01). This 10-year difference in MRD and all-cause costs was mostly driven by the higher number of MRD hospitalizations observed in GPP cohort compared with PV cohort. Table II presents the details on healthcare utilization and corresponding costs for our 2 cohorts. Over the 10-year time period, higher all-cause inpatient mortality was observed among patients with GPP compared with patients with PV (9.2% vs 7.3%; *P* = .01). Supplementary Table I (10-year costs available via Mendeley at https://data.mendeley.com/datasets/cg2zzmtyd2/1) and II (10-year mortality available via Mendeley at https://data.mendeley.com/datasets/cg2zzmtyd2/1) present the details of the multivariable regressions.

**Table II:** Prevalence analysis: 10-year mean (SD) health care resource utilization and costs, MRD versus all-cause admissions

Outcome	MRD∗ admissions			All-cause admissions		
	GPP (N = 607)	PV (N = 24,828)	*P* value†	GPP (N = 607)	PV (N = 24,828)	*P* value†
1. No. of hospitalizations per case	0.24 (0.50)	0.02 (0.16)	<.01	3.70 (6.32)	2.89 (4.59)	<.01
No. of hospitalizations amongst those with at least 1 hospitalization	1.11 (0.40)	1.14 (0.69)	.95	4.97 (6.88)	4.44 (5.05)	<.01
No. of days in the hospital for those with inpatient stay	10.36 (18.70)	6.97 (13.66)	<.01	8.91 (12.87)	9.50 (21.7)	.16
Cost of hospitalization for those with inpatient stay	$9728 ($22,545)	$5811 ($10,850)	<.01	$9506 ($11,305)	$10,696 ($16,894)	.31
2. No. of ED visits per case	0.49 (0.59)	0.44 (0.0.7)	.19	14.11 (24.43)	14.01 (27.79)	.49
No. of ED visits amongst those with at least 1 ED visit	1.08 (0.33)	1.18 (0.87)	.10	15.40 (25.13)	15.10 (28.57)	.32
Cost of ED visit for those with an ED visit	$205 ($162)	$158 ($68)	<.01	$282 ($165)	$308 ($182)	.01
3. No. of clinic visits per case	0.04 (0.25)	0.27 (4.15)	<.01	7.03 (41.08)	9.11 (51.22)	.13
No. of clinic visits amongst those with at least 1 clinic visit	1.30 (0.57)	4.17 (15.93)	.02	22.12 (70.65)	23.65 (80.41)	.84
Cost of clinic visit for those with clinic visit	$155 ($27)	$191 ($101)	<.01	$533 ($1050)	$524 ($937)	.87
4. No. of other NACRS visits	0.01 (0.11)	0.01 (0.15)	.34	4.58 (11.40)	5.50 (15.59)	.23
No. of other NACRS visits amongst those with at least 1 other NACRS visit	1.20 (0.45)	1.22 (0.69)	.86	8.34 (14.34)	9.09 (19.21)	.99
Cost of other NACRS visits for those with at least 1 other NACRS visit	$222 ($81)	$313 ($439)	.58	$781 ($670)	$755 ($732)	.70
Total costs (1 + 2 + 3 + 4)	$2393 ($11,410)	$222 ($1.828)	<.01	$49,695 ($111,582)	$42,574 ($83,960)	<.01

*ED,* Emergency department; *GPP,* generalized pustular psoriasis; *MRD,* most responsible diagnosis; *NACRS,* National Ambulatory Care Reporting System; *PV,* psoriasis vulgaris; *SD,* standard deviation.

∗ The MRD is the diagnosis (eg, GPP) determined to have been responsible for the greatest portion of the patient’s length of stay.

† *P* values based on multivariable generalized linear models adjusting for baseline characteristics (age, sex, and comorbidities).

### Incidence analyses

Out of 311 GPP incident cases between April 1, 2012, and March 31, 2017, 212 individuals (1.95 per million individuals) had an MRD code of GPP defining the index event. The corresponding number of incident cases for PV was 5979 (54.93 per million individuals). Aligned with our prevalence results, the incidence rates for GPP (3.68 per million individuals) and PV (158.84 per million individuals) were higher in Alberta compared with that in Ontario (GPP: 2.15 per million individuals; PV: 56.70 per million individuals) and when all provinces/territories were combined.

As shown in Table III, there was a higher proportion of women with GPP than those with PV (57.5% vs 45.3%; *P* < .01), and patients with GPP were older compared with those with PV (mean [SD] of 52.3 [15.6] years vs 48.3 [17.3] years; *P* < .01). Patients with GPP at the index had higher rates of comorbidities than patients with PV. The MRD index event defining a GPP or PV incident case was mostly related to an ED visit (70% for GPP vs 77% for PV; *P* < .01). The mean (SD) cost associated with the GPP index event was higher than that associated with PV index event ($2841 [$14,780] vs $275 [$1049]; *P* < .01) (Table IV).

**Table III:** Incidence analysis: baseline characteristics

Characteristic	GPP N = 212 (%)	PV N = 5979 (%)	*P* value
Women	122 (57.5)	2711 (45.3)	<.01
Mean age (SD)	52.3 (15.6)	48.3 (17.3)	<.01
Age categories (y)			<.01
18-25	7 (3.3)	567 (9.5)	
26-35	26 (12.3)	984 (16.5)	
36-45	33 (15.6)	997 (16.7)	
46-55	50 (23.6)	1199 (20.1)	
56-65	47 (22.2)	1107 (18.5)	
66-75	37 (17.5)	704 (11.8)	
≥75	12 (5.7)	421 (7.0)	
Province/Territory			<.01
Alberta	58 (27.4)	2505 (41.9)	
British Columbia	15 (7.1)	60 (1.0)	
Saskatchewan	ND	119 (2.0)	
Manitoba	ND	54 (0.9)	
Ontario	117 (55.2)	3092 (51.7)	
Nova Scotia, New Brunswick, Prince Edward Island, Newfoundland, and Territories	15 (7.1)	149 (2.5)	
Comorbidities			
Hypertension	42 (19.8)	756 (12.6)	<.01
Diabetes	25 (11.8)	514 (8.8)	<.01
Cigarette smoking	15 (7.1)	258 (4.3)	.05
Hyperlipidemia	8 (3.8)	147 (2.5)	.23
Myocardial infarction	5 (2.4)	94 (1.6)	<.01
Stroke	5 (2.4)	45 (0.8)	.37
Obesity	12 (5.7)	145 (0.8)	.01
Sleep apnea	ND	79 (1.3)	<.01
Depression	12 (5.7)	307 (5.1)	.48
Anxiety	ND	96 (1.67)	.73
Thyroid disorders	7 (3.3)	120 (2.0)	.19
Atopic dermatitis	ND	68 (1.1)	.19
Gout	11 (5.2)	119 (2.0)	<.01
Psoriatic arthritis	ND	36 (0.6)	.14

*GPP,* Generalized pustular psoriasis; *ND,* nondisclosable due to cell count less than 5; *PV,* psoriasis vulgaris; *SD,* standard deviation.

**Table IV:** Incidence analysis: mean (SD) health care resource utilization and costs associated with index event∗

Outcome	GPP (N = 212)	PV (N = 5979)	*P* value†
1. No. of hospitalizations	0.25 (0.43)	0.02 (0.14)	<.01‡
No. of days in the hospital for those with inpatient stay	8.3 (11.4)	6.2 (6.7)	.09
Cost of hospitalization for those with inpatient stay	$10,956 ($28,545)	$5217 ($5224)	.01
2. No. of ED visits per case	0.70 (0.46)	0.77 (0.42)	<.01‡
Cost of ED visit for those with 1 ED visit	$206 ($139)	$160 ($70)	<.01
3. No. of clinics and other NACRS visits per case	0.06 (0.14)	0.21 (0.41)	<.01‡
Cost of clinic and other NACRS records for those with a visit	$183 ($64)	$204 ($193)	.24
Total costs (1 +2 +3): Mean (SD)	$2841 ($14,780)	$275 ($1049)	<.01

*ED,* Emergency department; *GPP,* generalized pustular psoriasis; *NACRS,* National Ambulatory Care Reporting System; *PV,* psoriasis vulgaris; *SD,* standard deviation.

∗ Index event defined by the most responsible diagnosis (MRD) of GPP (GPP cohort) or PV (PV cohort). MRD is the diagnosis determined to have been responsible for the greatest portion of the patient’s length of stay.

† *P* values based on multivariable generalized linear models adjusting for baseline characteristics (age, sex, and comorbidities).

‡ *P* values based on multivariable logistic regressions adjusting for baseline characteristics as individuals had 0 or 1 event.

The cumulative 3-year mean (SD) MRD costs for patients with GPP and those with PV were $3477 ($14,979) and $503 ($2267) (*P* < .01), respectively. When health care resource utilization was included independent of the reasons leading to admissions/visits, the 3-year cumulative cost for incident cases was $17,665 ($49,219) for patients with GPP compared with $10,109 ($30,212) for those with PV (*P* < .01) (Table V). Three-year mortality was 5.2% for GPP incidence cases, and 2.1% for PV incidence cases (*P* = .03). The details of the index event costs, 3-year costs, and mortality multivariable regressions are presented in Supplementary Tables III (index costs), IV (3-year costs), and V (3-year mortality), available via Mendeley at https://data.mendeley.com/datasets/cg2zzmtyd2/1.

**Table V:** Incidence analysis: 3-year mean (SD) health care resource utilization and costs associated post index event, MRD∗ versus all-cause admissions

Outcome	MRD∗ admissions			All-cause admissions		
	GPP (N = 212)	PV (N = 5979)	*P* value†	GPP (N = 212)	PV (N = 5979)	*P* value†
1. No. of hospitalizations	0.36 (0.58)	0.03 (0.20)	<.01	1.23 (2.02)	0.62 (1.58)	<.01
No. of hospitalizations for those with at least 1 hospitalization	1.11 (0.43)	1.12 (0.42)	.80	2.51 (2.25)	2.29 (2.33)	.19
No. of days in the hospital for those with inpatient stay	7.84 (10.06)	6.62 (9.44)	.22	7.71 (8.60)	7.74 (12.74)	.21
Cost of hospitalization for those with inpatient stay	$9436 ($24,913)	$5530 ($6518)	.01	$9118 ($14,793)	$9560 ($16,015)	.98
2. No. of ED visits	0.76 (0.57)	0.88 (0.69)	.10	5.86 (10.52)	5.89 (12.43)	.83
No. of ED visits amongst those with at least 1 ED visit	1.09 (0.35)	1.13 (0.56)	.59	6.50 (11.0)	6.53 (13.00)	.99
Cost of ED visit for those with 1 ED visit	$214 ($139)	$163 ($66)	<.01	$276 ($138)	$256 ($144)	.50
3. No. of clinics and other NACRS visits	0.06 (0.26)	1.0 (8.08)	<.01	3.33 (11.15)	6.34 (22.72)	<.01
No. of clinics and other NACRS visits amongst those with at least 1 visit	1.08 (0.29)	4.71 (16.99)	.02	7.43 (15.82)	12.21 (30.25)	<.01
Cost of clinic and other NACRS visits for those with a visit	$183 ($64)	$204 ($191)	.27	$674 ($593)	$529 ($583)	<.01
Total Cost (1 +2 +3)	$3477 ($14,979)	$503 ($2267)	<.01	$17,665 ($49,219**)**	$10,109 ($30,212)	<.01

*ED,* Emergency department; *GPP,* generalized pustular psoriasis; *MRD*, most responsible diagnosis; *NACRS,* National Ambulatory Care Reporting System; *PV,* psoriasis vulgaris; *SD,* standard deviation.

∗ The most responsible diagnosis (MRD) is the diagnosis (eg, GPP) determined to have been responsible for the greatest portion of the patient’s length of stay.

† *P* values based on multivariable generalized linear models adjusting for baseline characteristics (age, sex, and comorbidities).

## Discussion

This is the first Canadian study and one of the few international studies documenting the burden of GPP. Based on Canadian population-based data for hospitalizations and visits to ED and hospital-/community-based outpatient clinics, our results indicate that the prevalence and incidence rates were the highest in Alberta at 5.4 per million individuals and 3.7 per million individuals, respectively, due to 100% reporting in this province. With GPP costs higher than PV costs by a factor ranging from 1.2 (all-cause costs) to 10.8 (MRD costs) in our 10-year prevalence analysis and from 1.7 (all-cause costs) to 6.9 (MRD costs) in our 3-year incidence analysis, the results demonstrate that patients with GPP experience a higher economic burden compared with patients with PV in Canada. In our 2 sets of analyses (MRD vs all-cause cost), this difference was mostly driven by higher hospitalization rates among patients with GPP. In addition, all-cause inpatient/ED mortality was also higher in the GPP population in our prevalence and incidence analyses. Our study results also showed that only a small part of the health care resource use and associated costs were attributable to an MRD of GPP or PV compared to all-cause admissions, which highlights the burden associated with GPP or PV complications or medical comorbidities in these patients.

Although comparisons with other studies may be difficult due to differences in design, data availability, and health care settings, our results are aligned with the emerging literature on the burden of GPP. The prevalence rates found in our study based on Canadian hospitalizations or visits to the ED or hospital-/community-based outpatient clinics (2.8-5.4 per million individuals) are similar to those derived from a study conducted in the same country (province of Ontario only)^12^ and a survey of a hospital dermatologic unit in France (1.8 per million individuals).^4^ Higher prevalence rates (7.5 to 122 per million habitants) have been reported in studies having access to physician data to identify patients with GPP.^4^^,^^6^^,^^11^ The incidence rates of our study (1.95 per million individuals) are also comparable with previous incidence data from France (0.6 per million individuals)^4^ and Sweden (4.2 to 8.2 per million individuals).^6^ Aligned with previous findings from Sweden^10^ and United States,^9^ this study highlights the higher economic burden of GPP than that of PV. Our study results differentiating health care resource utilization and costs directly attributable to GPP compared to other reasons were also consistent with the findings from Sweden, which showed that only 3% of Swedish patients with GPP were hospitalized due to GPP compared to 22% for any reasons.^10^ The mortality rates found in our study were also aligned with that of other studies.^8^

However, there are several limitations associated with our study. First, like all studies using administrative data, there is a risk of miscoding or underreporting the conditions under study. Second, another major limitation in CIHI data is that reporting a visit to ED and hospital-/community-based outpatient clinic data is not mandatory in all Canadian provinces/territories, and we may have underestimated the number of GPP admissions. To address this challenge, we conducted a sensitivity analysis using data from Ontario, where 100% of ED visits are reported in NACRS and Alberta for which reporting of ED and community clinics is mandatory. Third, we did not have access to patient-level physician billing data (eg, from dermatologists) to identify patients with GPP and those with PV diagnosed in the community or medications prescribed to patients with GPP and those with PV. Fourth, the cause of mortality as well as mortality in the community were also not available in our study data. Finally, individuals with an MRD of GPP were most likely admitted to the hospital or ED due to flareups, but we did not have any information on the intensity of the flareups leading to these admissions.

## Conclusion

Our results indicate that patients with GPP incur a higher human and economic burden than patients with PV, mostly due to a higher hospitalization rate among patients with GPP. Nonetheless, given the study limitations, our results represent an underestimation of the true burden of GPP. Future areas of research include the need to determine the burden of GPP in outpatient settings (eg, through physician billings and prescription drug databases) and to document the impact of GPP on quality of life, work/school productivity, as well as out-of-pocket expenditures, and caregiver burden.

## Conflicts of interest

McMaster University (Jean-Eric Tarride as Principal Investigator) has received a grant from Boehringer Ingelheim for this research. Dr Tarride has received honoraria from AbbVie, Amgen, AstraZeneca, Bayer, Edwards Lifesciences, Janssen, Lilly, Leo Pharma, Merck, Novartis, Novo Nordisk, Pfizer, Roche, and Takeda outside the scope of this paper. Dr Vimal H. Prajapati has been an advisor, consultant, and/or speaker for AbbVie, Actelion, Amgen, Aralez, Arcutis, Arena, Aspen, Bausch Health, Boehringer Ingelheim, Bristol Myers Squibb, Celgene, Cipher, Concert, Eli Lilly, Galderma, GlaxoSmithKline, Homeocan, Incyte, Janssen, LEO Pharma, L’Oreal, Medexus, Novartis, Pediapharm, Pfizer, Sanofi Genzyme, Sun Pharma, Tribute, UCB, and Valeant; he has also served as an investigator for AbbVie, Amgen, Arcutis, Arena, Asana, Bausch Health, Boehringer Ingelheim, Bristol Myers Squibb, Celgene, Concert, Dermavant, Dermira, Eli Lilly, Galderma, Incyte, Janssen, LEO Pharma, Nimbus Lakshmi, Novartis, Pfizer, Regeneron, Reistone, Sanofi Genzyme, UCB, and Valeant. Dr Charles Lynde has been a speaker and/or consultant to AbbVie, Altius, Amgen, Aralez, Arcutis, Bausch Health, Bayer, Boehringer Ingelheim, Bristol Myers Squibb, Celgene, Cipher, Dermavant, Devonian, Eli Lilly, Fresnius Kabi, Galderma, GSK, Innovaderm, Intega Skin, Janssen, Kyowa Kirin, La Roche Posay, LEO Pharma, L'Oreal, Medexus, MedX, Merck, Novartis, P&G, Pediapharm, Pfizer, Regeneron, Roche, Sanofi Genzyme, Sandoz, Sentrex, TEVA, Tribute, UCB, Valeant, Viatris, Volo Health; he has also been a principal investigator for AbbVie, Acleryin, Amgen, Aralez, Arcutis, Bausch Health, Bayer, Boehringer Ingelheim, Bristol Myers Squibb, Celgene, Cipher, Concert, Dermavant, Devonian, Eli Lilly, Galderma, GSK, Innovaderm, Janssen, Kyowa Kirin, LEO Pharma, L'Oreal, Merck, MoonLake, Pediapharm, Pfizer, Regeneron, Roche, Sanofi Genzyme, Tribute, UCB, Valeant. Gord Blackhouse has no conflicts of interest to declare.
